# Testing for Human Papillomaviruses in Urine, Blood, and Oral Specimens: an Update for the Laboratory

**DOI:** 10.1128/jcm.01403-22

**Published:** 2023-07-13

**Authors:** Mario Poljak, Kate Cuschieri, Laia Alemany, Alex Vorsters

**Affiliations:** a Institute of Microbiology and Immunology, Faculty of Medicine, University of Ljubljana, Ljubljana, Slovenia; b Scottish HPV Reference Laboratory, Royal Infirmary of Edinburgh, Edinburgh, Scotland, United Kingdom; c Cancer Epidemiology Research Program, Catalan Institute of Oncology, L’Hospitalet de Llobregat, Barcelona, Spain; d Centro de Investigación Biomédica en Red: Epidemiología y Salud Pública (CIBERESP), Instituto de Salud Carlos III, Madrid, Spain; e Centre for Evaluation of Vaccination, Vaccine & Infectious Disease Institute, University of Antwerp, Antwerp, Belgium; Vanderbilt University Medical Center

**Keywords:** HPV, urine, blood, oral specimens, cervical cancer, oropharyngeal cancer

## Abstract

Twelve high-risk alpha human papillomavirus (HPV) genotypes cause approximately 690,000 cancer cases annually, with cervical and oropharyngeal cancer being the two most prominent types. HPV testing is performed in laboratory settings for various applications of a clinical, epidemiological, and research nature using a range of clinical specimens collected by clinicians or by individuals (self-collected specimens). Here, we reflect on the importance and justification of using the right test for the right application and provide practical updates for laboratories either participating in or anticipating involvement in HPV testing in three specimen types, namely, urine, blood, and oral specimens, which are considered “alternative” specimens by many. In addition to clinician-collected cervical samples and self-collected cervicovaginal samples, first-void urine is emerging as a credible specimen for HPV-based cervical cancer screening, triage of HPV screen-positive women, monitoring HPV vaccine impact, and HPV testing in groups for which a less invasive sample is preferred. Detection of cell-free DNA (including HPV DNA) in blood has great promise for the early detection of HPV-attributable oropharyngeal cancer (HPV-AOC) and potentially other HPV-driven cancers and as an adjunct prognostic marker in long-term tumor surveillance, including treatment response. The moderate sensitivity of HPV testing in oral rinses or swabs at HPV-AOC diagnosis prevents its use in HPV-AOC secondary prevention but represents a promising prognostic tool in HPV-AOC tertiary prevention, where the HPV persistence in oral rinses throughout treatment may predict early HPV-AOC recurrences and/or the development of secondary HPV-AOC. The increasing sophistication of specific collection devices designed for alternative samples and the enhanced precision of novel molecular technologies are likely to support the evolution of this field and catalyze potential translation into routine practice.

## INTRODUCTION

Human papillomaviruses (HPVs) have a remarkably stable DNA genome and are classified by the homology of their genome into five genera (*Alpha*-, *Beta*-, *Gamma*-, *Mu*-, and *Nupapillomavirus*), several species, and numerous genotypes (1, 2). Genotypes are numbered chronologically in order of characterization, and 223 distinct HPV genotypes were officially recognized as of the end of June 2023 (https://www.hpvcenter.se/human_reference_clones/) (3). The 12 HPV genotypes from the *Alphapapillomavirus* genus with highest oncogenic potential are often referred to as high-risk HPV genotypes (hrHPVs) and are as follows: HPV-16, -18, -31, -33, -35, -39, -45, -51, -52, -56, -58, and -59 (3, 4).

Most hrHPV infections follow a subclinical course, but some persistent infections are etiologically linked with benign and malignant lesions of the epithelia (4, 5). Human cancers attributable to hrHPVs represent 4.5% of all cancers worldwide (8.8% and 0.9% of all cancers in women and men, respectively), with approximately 690,000 new cancer cases identified each year (6). hrHPVs cause virtually all cervical and anal cancers, a substantial proportion of vaginal cancers, and a component of oropharyngeal, penile, and vulvar cancer (5, 6). Consequently, laboratory detection of HPV to support screening for (or management of) associated disease has been the subject of research and development since the 1980s, of which some has translated into large-scale screening practice. Furthermore, general improvements in the precision of molecular testing and in our understanding of the natural history of HPV infection have ensured that the laboratory detection of HPV is still a highly dynamic field.

In this review, we reflect on the importance of “tailoring” the HPV test to the application and discuss the following three key and expanding areas in the laboratory detection of HPV: testing of urine, testing of blood, and testing of oral samples. Implications of this expansion for the detection and management of the two most common hrHPV driven cancers, namely, cervical and oropharyngeal, are our focus.

## THE BURDEN OF CERVICAL AND OROPHARYNGEAL CANCER AND THE IMPORTANCE OF EVIDENCE-BASED INTERVENTIONS

Cervical cancer, with 570,000 new annual cases worldwide, is the hrHPV-driven cancer associated with the greatest morbidity and mortality (6). Because hrHPVs are found in nearly all cases of cervical cancer, the etiological association between persistent hrHPV infection and this cancer is very strong, consistent, specific, and universal, making cervical cancer a highly credible target for public health preventive interventions, including through vaccination and screening. Although primary prevention via prophylactic HPV vaccination is a key tool for the elimination of cervical cancer (and other HPV-related cancers), the secondary prevention of cervical cancer via sensitive HPV-based screening using molecular tests is crucial and will be necessary for at least the next 50 years and will significantly accelerate the pace of cancer reduction (7–9). Increased access to HPV-based screening in “hard to reach” populations, including those in low- and middle-income countries will be essential if the goal of cervical cancer elimination is to be reached (7). The use of self-taken samples, including urine samples, is a clear way to support equitable access as will be discussed.

Oropharyngeal cancer is the second most common hrHPV-related human cancer, which is traditionally associated with chronic exposure to tobacco, heavy alcohol consumption, and deprivation (10). However, in the last 2 to 3 decades, hrHPV has emerged as an etiological agent for a subset of oropharyngeal cancer (6). Although the attributable fraction of HPV-associated oropharyngeal cancer varies according to geography, in many settings, it has increased markedly (11, 12). HPV-attributable oropharyngeal cancers (HPV-AOCs) show specific epidemiological, clinical, and molecular features that clearly differentiate them from those linked to tobacco and alcohol, including significantly improved prognosis (13). This difference is recognized in the recently updated tumor, node, metastasis (TNM) staging rules, which acknowledge the HPV-associated and HPV-independent pathway to oropharyngeal cancer (14). In 2018, a total of 140,000 new cases of oropharyngeal cancers were recorded worldwide (6). While the natural history of cervical cancer is relatively well understood and usually involves a preinvasive phase, which is detectable on screening, this is not the case for HPV-AOC. Thus, screening for HPV-AOC does not exist routinely, and patients generally present with clinical symptoms, frequently with advanced disease. Given the morbidity and increased incidence of this disease research to support improved prevention, screening and management strategies are areas of intense activity; laboratory-based biomarkers and tools to support the diagnosis and risk stratification are a cornerstone of this activity (15, 16).

## PRINCIPLES OF HPV TESTING—CHOOSING THE RIGHT TEST FOR THE RIGHT APPLICATION

While HPV testing is relevant for population-based screening of the healthy population, increasing evidence suggests it may be relevant for individual prognostication in the patient population (15). To serve both healthy and patient populations, a range of biological specimens (biospecimens) and HPV tests with different performance characteristics are required (17, 18). The characteristics of HPV tests used and the required specimen types differ considerably depending on whether the application for use serves a screening, diagnostic, epidemiological, or research objective (17, 18).

In routine clinical laboratory settings, testing for hrHPV is used most frequently as a screening tool in HPV-based primary cervical cancer screening programs (18). For this purpose, HPV testing must be performed using validated molecular tests with clinical sensitivity and specificity calibrated to the detection of high-grade cervical intraepithelial lesions rather than minute quantities of virus (17, 18). Until recently, this testing was performed almost exclusively using cervical samples collected by a trained health care worker (19, 20).

Compared with HPV screening tests, HPV assays required for epidemiology, including vaccine impact monitoring, have different characteristics with respect to analytical performance, HPV genotype range, and the biospecimen applied (5, 17, 18). For epidemiological purposes, archived specimens with partially degraded nucleic acids are often required, e.g., formalin-fixed, paraffin-embedded tissues. Tests suitable for archived material require higher analytical sensitivity, absolute analytical specificity, precise type-specific resolution of several HPV genotypes without cross-reactivity (leading to false-positive calls), and frequently an expanded or alternative composition of targeted HPV genotypes (17). A composition that includes additional HPV genotypes is clearly important for determining vaccine impact on low-risk genotypes (included in the HPV vaccine) and for investigating potential genotype replacement.

Similarly, HPV assays used for individual prognostication at the time of diagnosis and for monitoring treatment success require high analytical sensitivity and specificity, including in the context of head and neck disease (15). As will be discussed, tumor tissue is the specimen type used most frequently for determining the HPV status of oropharyngeal cancer, whether through PCR-based detection of HPV DNA or HPV mRNA *in situ* hybridization for viral sequences or (more commonly in routine settings) immunohistochemistry for an HPV-associated biomarker (p16^INK4a^) (21). While *in situ* hybridization and immunohistochemistry allow for the detection and identification of HPVs in topographical relation to their pathological lesions, its sensitivity can be lower than that of PCR (21, 22). Note, that when testing tissue specimens, strict anticontamination procedures are required at all levels—including meticulous histologic sectioning to prevent false-positives, a “sandwich” sectioning method for lesion verification, and optimized nucleic acid extraction protocols for HPV (21–23).

In line with this information, when it comes to HPV testing in blood, where virus quantities may be low and labile, highly sensitive PCR-based and alternative amplification approaches confer dividends on performance (24, 25). Digital droplet PCR (ddPCR) or partitioning PCR uses microfluidics to divide template DNA into many individual reactions (or droplets), which are then amplified separately. The fraction of PCR-positive droplets/reactions (out of the total number) is used to generate an absolute quantity. Whereas quantitative PCR (qPCR) generally offers one data point per reaction, ddPCR can offer several thousand and provides a sensitivity and precision that can be suitable for low-copy targets, particularly for early-stage disease (24). Its increased application is evident in the substantial number of publications in the last 3 years. Next-generation sequencing (NGS) has also been used for the detection of both HPV and cellular biomarkers in tissue and blood, and this technology allows precision and sensitivity for target detection and also offers possibilities for biomarker discovery depending on how it is applied (26).

Oral rinse and gargles using sterile saline or alcohol-based mouthwash are used most frequently for sampling the oral cavity or oropharynx for epidemiological and research purposes (27). Again, HPV tests required for these applications require high sensitivity, and the general acceleration of molecular diagnostics with improved sensitivity (including ddPCR and NGS) is likely to enhance this field (21).

As mentioned above, this review summarizes recent evidence and provides updates for the laboratories already participating or anticipating involvement in testing for HPV using the following three specimen types: urine, blood, and oral specimens. The three specimen types targeted by this review are considered “nonstandardized,” “alternative,” or “extragenital” by many; this language is not accepted uniformly, and the main dispute is whether urine is an “extragenital” or “genital” specimen type.

## HPV TESTING IN URINE

### HPV self-sampling.

To improve equitable access to screening, women in many cervical cancer screening programs or projects are now given the option of providing a self-collected sample (28–30). Self-taken samples for HPV testing have a similar accuracy to that of clinician-collected samples for the detection of high-grade cervical intraepithelial lesions when a validated PCR-based assay is used (20). In most self-sampling scenarios, women collect the self-sample by inserting a swab, brush, or more complicated collection device into the vagina to collect cervicovaginal secretions (containing exfoliated epithelial cells and debris of disintegrated cells) by turning or rubbing or through washing or lavage (21). However, urine as the least invasive self-collected sample has strong potential for use in self-sampling exercises for screening and epidemiological applications.

### Background.

Our understanding regarding the use of urine as a valid specimen for testing for HPV has evolved substantially in the last decade (Table 1). In the early days of investigation, major technical and interpretational challenges were identified and suboptimal analytical and clinical sensitivity were observed particularly when urine was compared with clinician-collected cervical samples (31). The mechanism through which HPV DNA “contaminated” urine was poorly understood and was a major limitation for advancing the field. An initial review on the performance of urine as a biospecimen for HPV testing demonstrated that the conflicting results obtained in early studies were largely a consequence of a lack of standardization and an imprecise broad definition of the term urine (31). In certain pioneering papers, investigators did not differentiate between the different “types” of urine, for example, initial-stream (first-void), midstream, total-void, any-void, first urine of the day, or urine collected later in the day. In addition, many investigators did not realize the considerable difference between female and male urine specimens and (wrongly) expected equivalent performance in both sexes.

**TABLE 1:** What is known, knowledge gaps, and how far are we away from implementation of first-void urine, blood and oral specimens as routine samples for HPV testing^*a*^

Sample type	What is known	Knowledge gaps	How far away are we from implementation as a routine specimen for HPV testing
Urine	First-void urine contains considerably more epithelial cells and their debris and consequently higher concentrations of both HPV and human DNA than those of subsequent void fractions. When using first-void urine, it is important to consistently use sample collection/transport medium containing urine-conservation preservative to stabilize HPV DNA to prevent its degradation by nucleases. If HPV testing is performed using clinically validated PCR-based HPV DNA assays and on appropriately collected and preserved first-void urine, similar clinical accuracy (CIN2+, CIN3+) as for/on self-collected cervicovaginal or clinician-collected cervical samples can be obtained. First-void urine sampling, using a validated collection device, is well accepted by women, is convenient, is noninvasive, and allows home collection of a sample for cervical cancer screening. First-void urine is a valuable source of other female reproductive and genital tract biomarkers, such as methylation markers, providing opportunities for triage of HPV-screen positives using (the same) self-collected sample. First-void urine has great potential to provide information on the HPV vaccination status of the women. First-void urine is emerging as a credible specimen for HPV-based cervical cancer screening, triage of HPV screen-positive women, and monitoring of HPV vaccine impact and for HPV testing in groups where a less invasive sample is preferable (including transgender individuals).	Widespread clinical implementation of urine-based HPV tests has been hindered by the lack of commercial standardized HPV tests. As for self-collected cervicovaginal samples in general, also for first-void urine, no HPV assay approved by FDA, EMA, or other stringent regulatory authority is available on the market. However, several countries have already implemented HPV-based cervical cancer screening using home collected cervicovaginal samples as an off-label screening strategy. Urine samples have been used for a number of in certain regions in France as an off-label screening strategy. The whole workflow from urine sample collection to result interpretation needs further optimization and standardization, including the determination of optimal sample volume and cut-off value for the interpretation of results. Including an internal control to monitor the whole workflow may be beneficial. HPV testing of urine samples has still not been incorporated into fully integrated, automated, sample-to-result molecular analyzers that allow continuous loading of samples and high-throughput testing. Only one first-void urine collection device is currently commercialized. Since this device is more expensive than devices for self-collected cervicovaginal samples, more devices for first-void urine collection are needed on the market for further cost optimization. Preliminary evidence regarding the acceptance and cost-effectiveness of cervical cancer screening using urine vs cervicovaginal self-collected samples in population-based organized screening settings have been collected in the last years. Pilot randomized clinical trials on the topic are ongoing in France (CapU4 study) and Belgium (ScreenUrSelf study).	HPV assay(s) approved for cervical cancer screening by stringent regulatory authority using self-collected cervicovaginal samples and first-void urine are expected on the market in the next 3 yrs. HPV testing of first-void urine samples using fully integrated, automated, sample-to-result molecular analyzers that allow continuous loading of samples and high-throughput testing are expected in the next 3 yrs. Some countries in Europe and selected low- and middle-income countries are expected to adopt first-void urine and other self-collected samples as a valid alternative to clinician-collected cervical samples in their population-based organized screening programs in the next 5 yrs. Some countries in Europe and some low- to middle-income countries are expected to adopt first-void urine as the valuable source of female reproductive and genital tract biomarkers, such as methylation markers, for triage of HPV-screen positives in the next 3 yrs. Novel anti-HPV assay(s) with enhanced analytical sensitivity will enable the collection of valid information regarding natural infection-induced anti-HPV antibodies in/from urine samples in the next 3 yrs.
Blood	Tumors can shed nucleic acid fragments into the circulating blood of patients; detection of these fragments is technically possible, particularly when using assays with high precision and sensitivity. Detectable nucleic acid fragments include ctDNA, miRNA and/or circulating viral DNA. These fragments are sometimes referred to collectively as “cell-free DNA” or “liquid biopsies.” Although HPV-AOC is rising, no validated screening strategy is currently available. To improve the screening and management of HPV-AOC, the following two approaches have been explored, of which both utilize blood as a biospecimen: (i) detection of antibodies against HPV-16 E6 protein and (ii) liquid biopsies or the detection of cell-free DNA (including HPV DNA). Antibodies against HPV-16 E6 protein are detectable 10 yrs prior to the diagnosis of HPV-AOC. Those individuals who are antibody positive are at significantly greater risk of HPV-AOC. Detection of HPV in liquid biopsies is indicative of disease recurrence posttreatment in HPV-AOC. Nucleic acid fragments in liquid biopsies have a short half-life; swift processing is thus key to prevent degradation. Improved (blood) capture and processing protocols in addition to a greater application of highly sensitive detection techniques, such as digital droplet PCR (ddPCR) and next-generation sequencing (NGS), are likely to enhance the performance of the technology overall.	Due to a poor positive predictive value as a result of the low burden of HPV-AOC in the general population, the lack of an accepted algorithm for follow-up of positive serology cases, and the need for further optimization of the assay(s), the potential of anti-HPV-16 E6 antibody testing for screening and early detection of HPV-AOC is unclear at present. Anti-HPV-16 E6 antibody presence in blood may be useful for annotation of the HPV status of oropharyngeal cancer in selected clinical scenarios and may serve as a potential prognostic marker of HPV-AOC, but greater research in these areas is warranted. Studies with larger sample sizes, optimized clinical protocols with clear longitudinal outcomes, and refined, consistent technical methodology are needed to better establish the clinical utility of liquid biopsies or the detection of cell-free DNA (including HPV DNA) in blood samples. A specific tumor signature for the component of HPV-unrelated oropharyngeal cancers (that can be detected consistently in blood) has so far proven elusive, which is likely due to the heterogeneity of tumors with a nonviral etiology. There is evidence to suggest that liquid biopsies may be suitable for the management of other HPV-associated anogenital cancers, including cervical and anal cancer, particularly in high-risk populations; however, larger follow up studies are needed to confirm a definitive clinical use case. Very little is known about the performance of liquid biopsy for the detection and prognosis of vaginal, vulvar, and penile disease. Greater research in this area is warranted.	It is likely that the first routine application of liquid biopsies in the HPV field will be for the monitoring of treatment response in patients with HPV-AOC. Private entities offer liquid biopsy testing (for HPV in blood) for the management of HPV-AOC, although it is not considered standard of care. Reports on the use of liquid biopsies to measure treatment response in longitudinal clinical trials compared with other more standard clinical modalities will be essential. These will help determine how the technology can be applied optimally to deintensify follow-up and are likely to report in the next 3–5 yrs. Significant improvements in molecular detection technology (and growing academic and commercial interest in the area) are likely to lead to standardized HPV assays for liquid biopsies in 1–3 yrs. Assays applicable to liquid biopsies already exist in the commercial sector for non-HPV targets and is likely to facilitate development.
Oral samples	HPV-AOC is a distinct disease, markedly different from oropharyngeal cancer etiologically associated with tobacco and alcohol at the following three levels: epidemiological, clinical, and molecular. Compared with cancers etiologically associated with tobacco and alcohol, HPV-AOC has a substantially better prognosis and superior response to treatment. The incidence of HPV-AOC is rising in many countries even surpassing the incidence of cervical cancer in some regions. Overall, 30% of oropharyngeal cancers are currently attributable to HPV, with substantial geographical variability in HPV-attributable fractions being as high as 80% in North America and northern Europe. Differentiation of HPV-AOC from HPV-non-attributable oropharyngeal cancers is currently a major clinical application of HPV testing. Annotation of the HPV status of oropharyngeal cancer is best ascertained by testing tumor tissue for the overexpression of p16^INK4a^ using immunohistochemistry ideally in combination with the presence of HPV DNA or HPV mRNA discerned either by PCR-based methods or *in situ* hybridization. Detection of HPV persistence in oral rinses and gargles using sterile saline or alcohol-based mouthwash (and/or by detecting cell-free DNA in blood) throughout treatment and posttreatment represent encouraging tools for monitoring response and for predicting disease recurrences after therapy. The use of preservative added immediately to the collection tube after oral sample collection improves the stability of the oral sample. Oral samples should be processed as close to collection as possible or alternatively immediately frozen at −20°C. Because HPV viral load in oral (and also blood) samples is significantly lower than that in genital sites, testing oral specimens requires the use of HPV tests with substantially higher analytical sensitivity.	The key knowledge gaps in the natural history of HPV-AOC that hinder the implementation of secondary prevention lie around the existence (or not) of HPV-related preneoplastic lesions and, if such lesions exist, whether they can be identified early enough to be treated in a safe, effective, and acceptable way to prevent development of HPV-AOC, as in the case of cervical cancer. No validated screening strategy for the secondary prevention of HPV-AOC aiming for early disease detection in the healthy population is available at present. Nontissue oral sample alternatives for differentiation of HPV-AOC from HPV-non-attributable oropharyngeal cancers, including (i) oral rinse and gargle using sterile saline or alcohol-based mouthwash and (ii) swabbing or brushing of surface of visible lesions, have been studied intensively in the past decade, but the moderate sensitivity limits their use as a valid diagnostic tool currently. The whole workflow from oral sample collection to result interpretation needs further optimization and standardization, including the determination of optimal sample volume and cut-off value for an interpretation of the results.	Standardized, clinically validated oral rinse and gargle collection protocols and standardized collection devices allowing reliable HPV testing are expected in the next 3 yrs. Commercial HPV assay(s) approved by stringent regulatory authority using oral samples are expected on the market in the next 5 yrs.

a Abbreviations: HPV, human papillomavirus; CIN2+, cervical intraepithelial lesion grade 2 or higher; CIN3+, cervical intraepithelial lesion grade 3 or higher; FDA, United States Food and Drug Administration; EMA, European Medicines Agency; ctDNA, circulating tumor DNA; miRNA, microRNA; ddPCR, digital droplet PCR; NGS, next-generation sequencing; HPV-AOC, HPV-attributable oropharyngeal cancer.

### Rationale for the preferable use of first-void urine for HPV testing in women.

**FIG 1:**
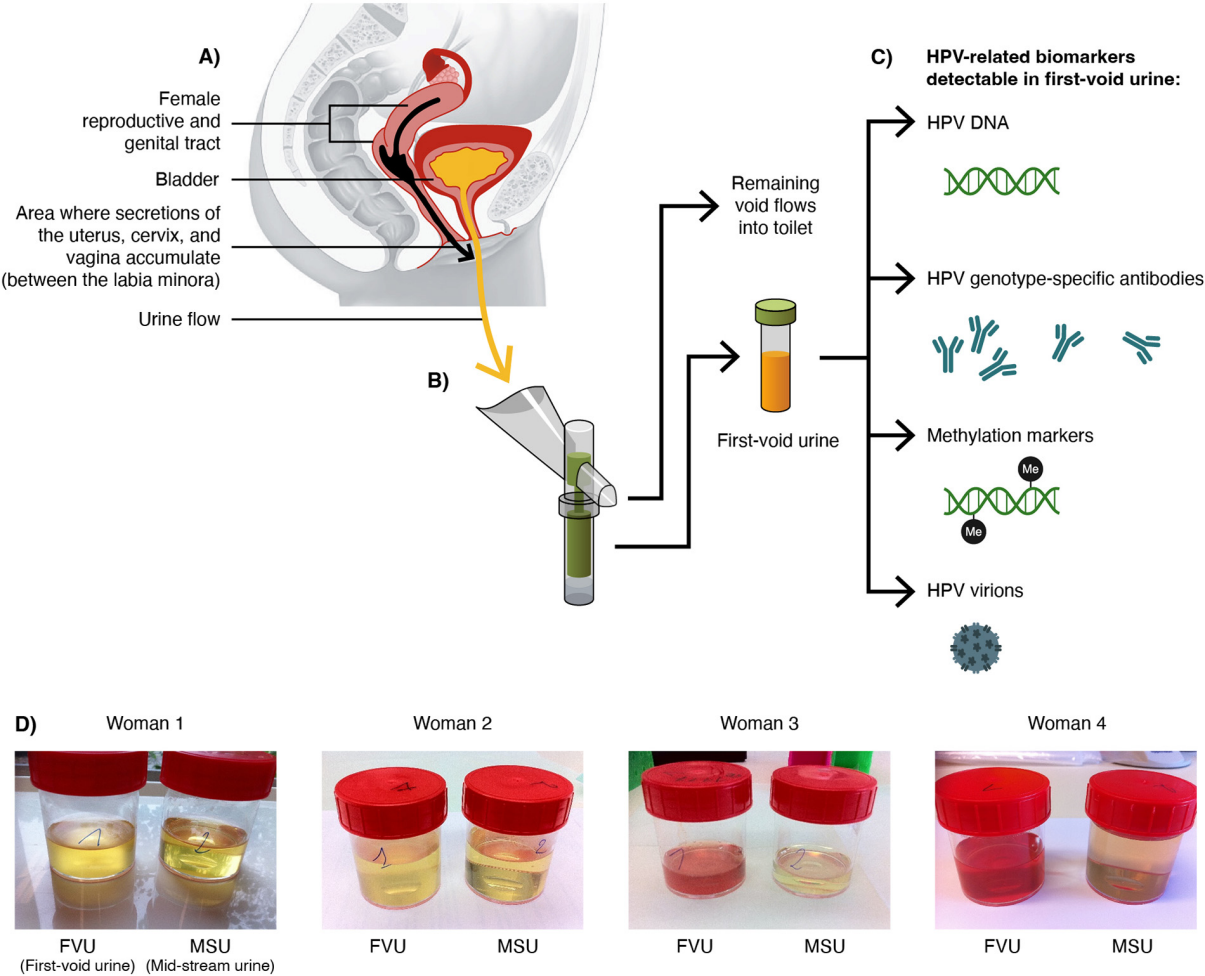
Rationale for the use of first-void urine as source of female reproductive and genital tract biomarkers and HPV-related biomarkers detectable in first-void urine. (A) Presents the anatomy of the female reproductive and genital tract and bladder, with the black arrow depicting the physiological movement of secretions of the uterus, cervix, and vagina subsequently mixed with debris of superficial cell layers of the epithelium covering the entire surface of the genital tract, exfoliated cells, and transudated or exudated immunoglobulins (mainly IgG). All secretions further drift and exit the vagina, where they are accumulated between the labia minora and around the urethra opening and flushed away by the urine originating from the bladder (yellow arrow). (B) Shows a first-void urine collection device (Colli-Pee; Novosanis, Wijnegem, Belgium) that can be prefilled with a urine preservative and is able to capture a prespecified volume of first-void or initial-stream urine without interrupting the urine void. Once the collection tube is filled, an outlet ensures that subsequent urine volume exits the device into the toilet. (C) Shows several relevant biomarkers detectable in first-void urine, as follows: (i) cell-free and cell-associated HPV DNA; (ii) transudated or excudated immunoglobulins, including HPV genotype-specific antibodies; (iii) methylated viral and human DNA; and (iv) HPV virions in the case of a productive HPV infection. (D) Shows selected examples of urine specimens collected by four out of eight women that captured their initial or first-void urine (FVU) and the subsequent void (often referred to as midstream urine [MSU]) directly into urine collection containers. In six of the eight women, macroscopically visible differences between FVU versus MSU samples due to substantially more debris derived from superficial layers of the epithelium, exfoliated cells, and genital tract secretions present in FVU were noticed. In the samples from women 3 and 4, who were menstruating at the time of sample collection, the FVU samples also contained menstrual blood visible with the naked eye compared with an almost transparent MSU sample(s).

When different types of urine specimens were assessed from a cohort of HPV-positive women, substantial differences were observed in the reported copy number of HPV and human DNA when comparing first-void (or initial-stream) urine versus the subsequent fractions (32). It is now generally agreed that first-void urine contains considerably more epithelial cells and consequently higher concentrations of both HPV and human DNA than subsequent void fractions. This observation is in fact consistent with an opinion forged in the early 1940s, where Traut and Papanicolaou (33) suggested that for the detection of cancer of the uterus a self-collected vaginal smear would be appropriate and could be used for routine diagnostic purposes. Their basic rationale for this suggestion was that all epithelial tissues have superficial cell layers that are subject to continuous exfoliation. This debris of epithelial tissues and exfoliated cells subsequently mix with the secretions of the uterus and cervix and descend into the vagina, explaining why they can be detected in a vaginal sample. Subsequent to this information came the realization that female genital secretions, including the debris of superficial cell layers, further descend and exit the vagina, where they are accumulated between the labia minora and around the urethra opening (Fig. 1). This natural biological cleaning process of the female genital tract is completed when a woman urinates and flushes away (mostly with first-void urine) said accumulated secretions. Thus, in the context of HPV testing, we should not categorize first-void urine as a urine specimen but rather consider it a mixture of genital tract secretions collected through urination. In Fig. 1, this important rationale is shown graphically, and four selected examples of macroscopically visible differences between first-void urine versus subsequent urine fraction(s) are presented.

The type of urine specimen or the choice of void fraction used for HPV testing does have a profound impact on the amount of HPV DNA present in the sample and, in turn, the probability of accurate HPV detection. The superiority of first-void urine for HPV testing was confirmed in the first meta-analysis on the subject, published in 2014, but without understanding the underlying reasons and mechanism (34). Surprisingly, although the fraction of the void exerted an impact on testing accuracy, as well as use of a tailored device to capture the initial void, no difference between (first-void) urine collected during first morning urination versus later in the day was observed (35, 36). Consequently, in applied projects where women are asked to collect urine for HPV DNA testing, they are asked not to wash their genitals thoroughly in advance of the sample and to wait at least 1 h after the previous urination before providing a first-void urine specimen for HPV testing (34).

### Other technical considerations.

Another important observation described in pilot experiments is the relative instability of HPV DNA in certain urine samples and the requirement for a preservative in the sample collection/transport media to enhance stability and minimize degradation. Even with well-established DNA extraction protocols, which have been found to garner a high yield and quality of HPV DNA in more cellular samples, significant reductions of HPV DNA in urine specimens have been observed in the absence of a preservative applied immediately after collection of first-void urine (32). This finding is consistent with the fact that, due to nucleases present in unpreserved urine, HPV DNA may become undetectable (32). The instability of HPV DNA in urine is its main drawback compared with cervical samples, where HPV DNA remains stable at room temperature for months. Therefore, it is important when using first-void urine to incorporate a sample collection system that includes urine-conservation preservative to stabilize HPV DNA. In addition, human DNA present in urine may not be optimal as an internal control for sample validity in urine because it can remain positive even if HPV DNA is degraded substantially, running the risk of false-negative results (32). Instead, the use of an external DNA control to monitor sample validity is recommended for urine (37).

### Use of urine in primary HPV-based cervical cancer screening.

By applying the “lessons learned” for the optimal capture and preservation described above, good analytic concordance between such first-void urine versus clinician-collected cervical samples has been demonstrated in pilot studies, with respect not only to the spectrum of HPV genotypes detected but also to genotype-specific viral load (38). Such studies clearly demonstrated the significant potential of first-void urine as a valid alternative to clinician-collected cervical and self-collected cervicovaginal specimens for HPV-based cervical cancer screening programs (Table 1). Subsequent to these pilot studies, clinical validation trials have been performed, showing positive results. The international validation of human papillomavirus assays and collection devices for HPV testing on self-samples (VALHUDES) diagnostic test accuracy study follows a design where the clinical performance of validated PCR-based HPV DNA assays on optimally collected and preserved first-void urine samples is compared with clinician-collected cervical samples, with the latter considered standard of care (39). Existing results are very promising, and a number of commercially available HPV DNA assays, including the US FDA-approved Onclarity HPV assay (BD Diagnostics, Sparks, MD) and Cobas 6800 HPV assay (Roche Molecular Systems, Branchburg, NJ), as well as the WHO-prequalified RealTime high-risk HPV test (Abbott Molecular, Des Plaines, IL), showed comparable clinical sensitivity and specificity for cervical intraepithelial neoplasia grade 2 or higher (CIN2+) using at-home self-collected first-void urine samples versus clinician-collected cervical samples (40–44). Compared with clinician-collected cervical samples, first-void urine is relatively easy to self-collect, is noninvasive, and allows home collection of a sample for cervical cancer screening. In addition, qualitative research comparing urine-based self-sampling to cervicovaginal self-sampling has shown that some women prefer urine sampling, and in a study by De Pauw et al. (42), substantially more women were confident they had performed the sampling correctly when providing a urine sample. However, the preference and affordability of a particular sampling method are important aspects that are clearly context specific.

One potential operational consideration for “scaling-up” urine-based screening is that, compared with cervical samples, HPV testing of urine samples has still not yet been fully integrated into automated, sample-to-result molecular analyzers that allow the continuous loading of samples and high-throughput testing.

### Use of urine for triage of HPV screen-positive women.

A further promising application when using self-collected samples, including first-void urine, is the potential to use the same sample to screen for hrHPV infection and to detect biomarkers that triage the risk of that infection to cause significant disease, such as DNA methylation markers (Fig. 1). Multiple studies have shown the high accuracy of DNA methylation assays as a triage tool in hrHPV-positive vaginal swabs for detecting high-grade lesions (45). DNA methylation assays have shown equivalent performance as cytology for detecting underlying CIN2+ lesions and a greater longitudinal negative predictive value for the absence of disease at follow-up than cytology-negative women (46). Recently, the clinical value of DNA methylation markers to discern clinically relevant disease was demonstrated in first-void urine samples (47, 48). This knowledge further supports self-samples, including first-void urine, as credible specimens for cervical cancer screening programs with the potential to support underserved women and avoid overreferral through molecular triage.

### Monitoring the impact of HPV vaccination using urine.

In addition to screening applications, first-void urine samples are a highly practical means to sample young individuals to ascertain the population-level impact of HPV vaccination. Indeed, in cohorts of young women, who may or may not yet be sexually active, urine sampling is generally well accepted. The large HPV vaccination monitoring impact trials performed in Rwanda and Bhutan targeting young women between the ages 17 and 22 showed that 94% of the 4,463 participants returned their (at-home) self-collected urine samples for HPV testing (49). In these trials, urine samples were self-collected by participants using a specific device (Colli-Pee; Novosanis, Wijnegem, Belgium) designed to collect the first 14 mL of first-void urine immediately into 7 mL of a urine-conservation medium while the subsequent urine volume exits into the lavatory (Fig. 1).

### Detection of anti-HPV antibody in urine.

As mentioned, a first-void urine specimen contains female genital secretions, including those harboring locally produced immunoglobulins or immunoglobulins transferred from blood by transudation or exudation (50). Perhaps unsurprisingly, it was thus confirmed that first-void urine also contains functional neutralizing anti-HPV antibodies, and a statistically significant correlation between the concentration of genotype-specific anti-HPV antibody in serum samples and in first-void urine samples was demonstrated (51). This information provides an opportunity to use a first-void urine sample to detect active HPV infection as well as immune status after HPV vaccination or after natural HPV infection (by testing for anti-HPV antibodies). During a productive HPV infection, new infectious viral particles are released from the outer layers of the epithelium and mix with the genital secretions. In HPV-vaccinated women, these genital secretions also contain vaccine-induced neutralizing anti-HPV antibodies that may bind to the infectious HPV viral particles. This process may substantially impact infectivity, help prevent transmission to a sexual partner, and reduce the chance of autoinoculation, or self-infection of other parts of the body (52). Because a mixture of the genital secretions can be collected easily using first-void urine, this specimen has significant potential for further research on the impact of HPV vaccination and may assist in defining a much-needed correlate of protection (the minimum concentration of anti-HPV antibody required to prevent transmission) especially in view of the recent implementation of one-dose HPV vaccination schedules.

### Use of urine for HPV testing in males.

In contrast to women, for whom a first-void urine specimen is a credible alternative to both clinician-collected and self-collected specimens, urine specimens collected from males (including first-void urine) are less appropriate and accurate for the detection of anogenital HPV infections because the anogential secretions “collected” by urination differ substantially due to anatomical differences (53). Consequently, a significantly lower HPV positivity rate and also smaller amounts of human DNA are reported in male first-void urine specimens than those collected from women.

### Use of urine for HPV testing in transgender individuals.

There are limited data on HPV prevalence, the natural history of HPV-related tumors, and screening options in transgender individuals due to the low acceptance rate of traditional sample collection approaches. A recent pilot study on 200 transgender individuals showed a high acceptance rate (98.5%) of first-void urine self-collected using the Colli-Pee FV-5000 collection device for HPV testing, providing a significant opportunity for natural history, epidemiological, and screening studies in this hard-to-reach population (54).

## TESTING FOR HPV IN BLOOD

### Background.

Blood as a specimen has attracted significant recent attention for the detection and management of HPV-associated disease because it can contain detectable biofragments that may be indicative of underlying or recurrent neoplastic disease (25, 55). Such fragments include microRNA (miRNA), circulating tumor DNA (ctDNA), and/or circulating HPV DNA (cHPV DNA), sometimes referred to collectively as “liquid biopsies” or “cell-free DNA.” In addition, the detection of antibodies against the HPV-16 E6 protein in blood has been explored extensively in the last decade as a potential screening, diagnostic, and prognostic tool for the early detection and monitoring of HPV-AOC (15, 56).

### The use of liquid biopsy for detecting HPV-AOC.

To date, the bulk of studies that have explored the utility of liquid biopsies for detecting HPV-associated disease have focused on oropharyngeal cancer, particularly using cHPV DNA as a target. As we will go onto describe, the mutational landscape of oropharyngeal cancer is complex, and so the determination of a core tumor target set to cover HPV-independent tumors generically has thus far proved elusive. In a study by Warlow et al. (57), the detection of HPV DNA in pretreatment plasma using ddPCR showed high (>90%) agreement with p16^INK4a^ and the HPV PCR status of the pretreatment (solid) biopsy samples in oropharyngeal cancer patients. The detection of cHPV DNA has also been shown to be indicative of disease recurrence posttreatment. In a study of over 100 HPV-AOC patients treated with curative intent, of the 87 patients that had undetectable cHPV DNA, none had recurrent disease during follow-up (median 23 months), whereas the sequential detection of cHPV in two posttreatment samples exhibited a positive predictive value of 94% (58). A recent meta-analysis on the performance of cHPV DNA in monitoring treatment response in 457 HPV-AOC patients showed a pooled diagnostic sensitivity and specificity of 65% (95% confidence interval [CI], 40 to 84) and 99% (95% CI, 96 to 99), respectively (59). This range reflects the heterogeneity of inclusion criteria, protocols, and detection technology summarized by the authors, who stated that “Larger sample sizes and the homologation of study protocols and methodology are needed to better establish its utility in the clinical practice” (59).

### The use of liquid biopsy for detecting cervical cancer.

After oropharyngeal cancer, liquid biopsies have been researched most extensively for cervical cancer studies. The targets evaluated have included cHPV DNA, the more common cancer mutations (e.g., PIK3CA), and host and viral methylation targets, including CADM1 and EPB41L3 as recently reviewed by Herbst et al. (60). Most studies in the cervical context have focused on the detection of invasive disease rather than preinvasive disease. Given that the extent of vascularization is likely to influence the secretion of relevant biomolecules into the blood, it is logical that the sensitivity of liquid biopsy for detecting CIN2 and CIN3 may be limited, although proof-of-concept studies exist to show it is possible, including for methylated targets (61). The 2020 meta-analysis assessed the diagnostic performance of cHPV DNA for detecting invasive cervical cancer across 10 different studies conducted between 2001 and 2017, and it reported a pooled sensitivity of 27% (95% CI, 24 to 30) (62). This modest sensitivity reflected a range of diagnostic approaches and techniques used with respect to chemistry, the biospecimen (serum versus plasma) target molecule, and HPV genotype coverage. Because cervical cancer is etiologically associated with a range of hrHPV genotypes (as described earlier), genotype coverage is not trivial. In common with the meta-analysis on oropharyngeal cancer (59), the authors called on the scientific community to optimize and standardize the technical approach. This endeavor seems worthwhile; although investment in biomarkers for cervical disease in the healthy screening population is of course essential, there is a comparative lack of research in the posttreatment population, which remains at a higher risk of recurrent disease up to 20 years after initial treatment (63, 64).

### Applications of liquid biopsy for other HPV-related cancers.

There are a smaller but growing number of studies on the use of liquid biopsy for detecting anal cancer; in a study of 57 patients with advanced anal cancer, HPV ctDNA was detected in 91.1% when an HPV-16 genotype-specific HPV assay was employed (65). In a more recent study in which the authors employed NGS technology for eight hrHPV genotypes, the sensitivity increased to 100% in 20 patients with histologically confirmed disease (26). Although the data on the use of liquid biopsies for managing anal disease are relatively scant, they are encouraging—particularly because this disease can be challenging to treat, and screening protocols are generally not well developed or established, even in high-risk populations. In addition, there are very few data on the feasibility and application of liquid biopsy for diagnosing and monitoring vulvar, penile, and vaginal cancer and its precursors; given the morbidity of these diseases and their potential for recurrence, this area would benefit from more attention.

### Technical challenges of liquid biopsy and opportunities for improvement.

**FIG 2:**
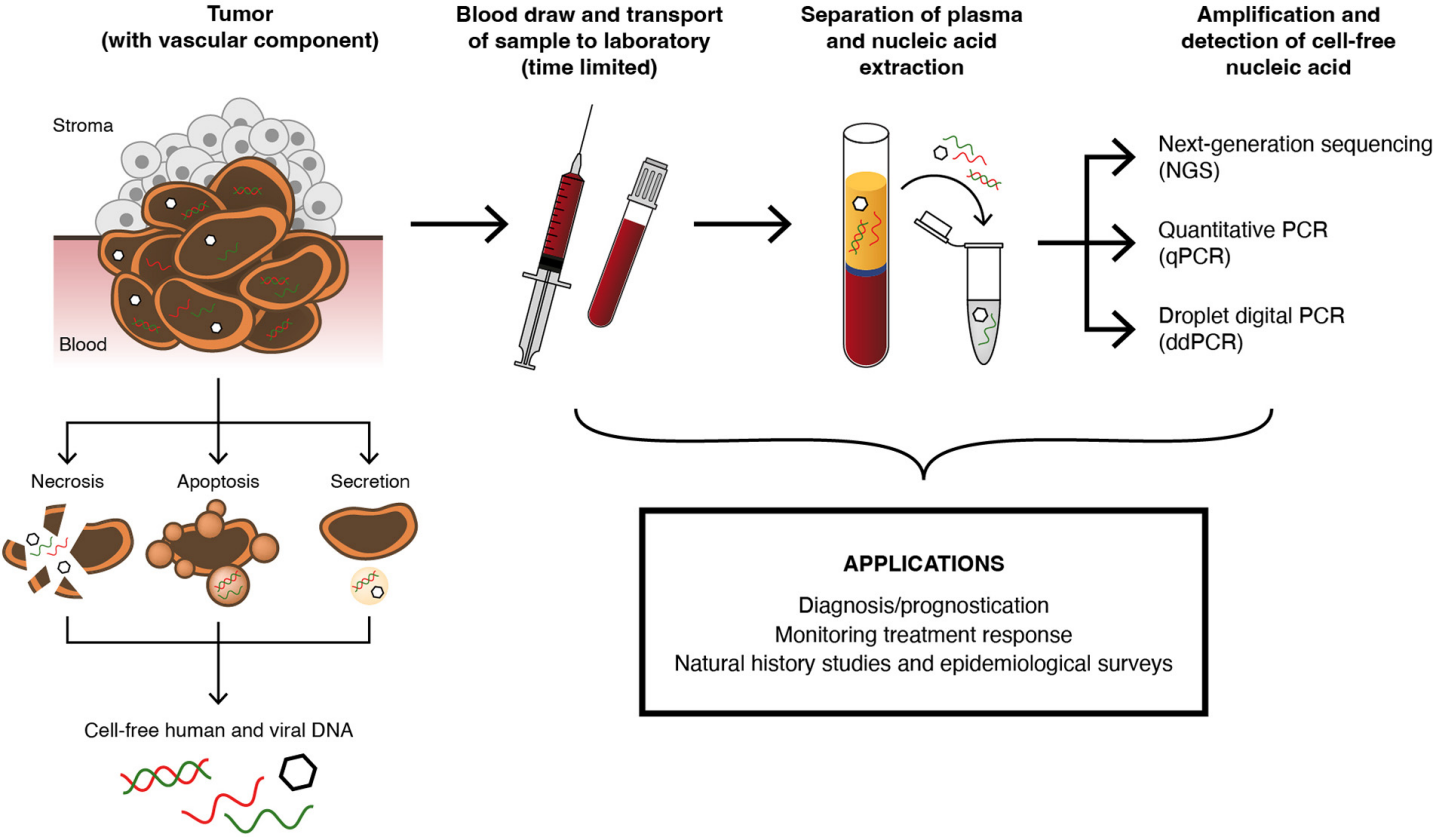
Process and rationale for the detection of cell-free DNA (including HPV DNA). Abnormal lesions and cancers with a vascular component contain nuclear material that can be shed into the blood through various pathways, including necrosis, apoptosis, and secretion. This material, referred to as cell-free DNA (cfDNA), can be double or single stranded, and the concentration and stability depend on a number of factors, including lesion size, extent of proliferation, and extent of vascularization. cfDNA can be host or viral in origin, and it is feasible for a lesion to exude both. After a blood draw and centrifugation, nucleic acid that contains cfDNA can be extracted from the plasma component. Given the short half-life of some cfDNA targets, efficient sample transfer to the laboratory for processing and extraction is important. cfDNA can be used to detect viral and/or host sequences, specific mutations, integration hallmarks, methylation targets, and microsatellite alterations. Although a range of amplification technologies, such as quantitative PCR (qPCR), have been used for detecting cfDNA, those methods that can offer high analytical sensitivity are valuable, such as next-generation sequencing (NGS) and droplet digital PCR (ddPCR). The translation of cfDNA into routine diagnostic laboratory practice is at a relatively early stage for HPV-associated disease, but it holds promise for various applications, including diagnosis/prognostication and monitoring treatment response. In addition, cfDNA determination is valuable in natural history studies and epidemiological surveys.

Circulating DNA can be single or double stranded and is generally secreted into the blood at an early stage of cancer development through mechanisms that include apoptosis or tumor necrosis (60) (Fig. 2). The target may have a short half-life of between 20 min and a few hours, which means that the capture of the sample and processing of the blood specimen should be well controlled and validated. Aspects to consider are the blood capture volume and collection tube type, transport conditions, maximum time from blood capture to separation of cell-free component (usually plasma), method for capture of plasma (including centrifugation conditions), volume-input for nucleic acid extraction, and method of nucleic acid extraction. It is notable that, although these conditions are important, particularly if liquid biopsy testing is to be integrated into routine service, this level of technical detail is frequently missing from published articles and may be part of the reason why there is variation in diagnostic performance between different laboratories. A similar lack of important technical detail in the published literature was also described in a recent systematic review of the detection of various urine biomarkers, including HPV (66).

After nucleic acid extraction from plasma, the extract may be frozen at −20°C prior to testing, but again, the impact of any longer-term storage on target deterioration should be quantified. The technology is likely to be supported by the increase in the availability of commercial kits, which have been optimized to extract circulating nucleic acid of high quality.

### The evolution of technology for liquid biopsy detection.

Diagnostic targets in liquid biopsies vary and reflect the virus, host, or both; with regard to the virus, the focus has often been only on HPV-16 and/or HPV-18, although there are examples of broader HPV panels (26). Methylated targets have also been investigated in liquid biopsies of patients suffering from HPV-associated cancers (59). The systematic reviews of oropharyngeal and cervical disease incorporate a range of technologies and reflect the molecular evolution of diagnostics in general (59, 62). Some of the earliest techniques applied were standard PCR or nested PCR followed by Southern blotting or agarose electrophoresis. These methods preceded the application of quantitative PCR (qPCR) and, more recently, NGS and ddPCR.

NGS can detect insertions, deletions, somatic single nucleotide polymorphisms, and gene copy number variations, and it has been applied in an exploratory manner to define biomarkers of interest in cancer patients and, in turn, to create cancer-target “panels,” including for cervical cancer patients (67). NGS also offers the opportunity for the design and application of a test that is specifically tailored to the mutation profile within an individual patient. This prospect is an exciting one, although bioinformatic and technical infrastructure clearly need to be in place; furthermore, NGS can be deployed in a simpler manner to detect viral sequences in blood (26).

Molecular developments in areas such as NGS and ddPCR will clearly help refine the technology and will support quality and consistency. The update of digital minimum information for publication of quantitative digital PCR experiments (dMIQE) is helpful in this regard (24). Although translation and assay commercialization of the end-to-end process of liquid biopsy is more developed for other cancers—for example, epidermal growth factor receptor (EGFR) mutation testing for lung cancer patients—the experience from such applications is also likely to enhance the HPV field (Table 1).

### Anti-HPV-16 E6 antibody as a screening, diagnostic, and prognostic marker of HPV-AOC.

In the last decade, several investigators have shown that anti-HPV-16 E6 antibody presence in blood may serve as a potential screening, diagnostic, and prognostic marker of HPV-AOC (68). A pioneering study showed that anti-HPV-16 E6 seropositivity was present up to 10 years before the diagnosis of HPV-AOC (69). In a recent systematic literature review and meta-analysis, the sensitivity of HPV-16 E6 seropositivity for underlying HPV-16-driven HPV-AOC was 83.1% (95% CI, 72.5 to 90.2) with a specificity of 94.6% (95% CI, 89.0 to 97.4) (68). However, due to a poor positive predictive value due to the low burden of HPV-AOC in the general population, the lack of an accepted algorithm for the follow-up of positive serology cases, and the need for further optimization of the assay(s), the potential of anti-HPV-16 E6 antibody testing for screening/early detection of HPV-AOC is unclear at present. The anti-HPV-16 E6 antibody has also been piloted for diagnostic purposes, e.g., annotation of the HPV status of oropharyngeal cancer in selected clinical scenarios, but greater research in this area is warranted (13, 15). A few studies have also explored the use of anti-HPV-16 E6 antibody titer dynamics as a prognostic marker of tumor recurrence in the tertiary prevention of HPV-AOC but with mixed and inconclusive results (15).

## TESTING FOR HPV IN ORAL SPECIMENS

### Rationale for detecting HPV in oral specimens.

As discussed, strong evidence exists to show that HPV-AOC is a distinct disease and is markedly different from oropharyngeal cancer etiologically associated with tobacco and alcohol at three levels, as follows: epidemiological, clinical, and molecular (13).

The incidence of HPV-AOC is rising in many countries and is even surpassing the incidence of cervical cancer in some regions (11, 12). Overall, 30% of oropharyngeal cancers are currently attributable to HPV, although with substantial geographical variability in HPV-attributable fractions (as high as 80% in North America and northern Europe) (70). Globally, more than 80% of HPV-AOC cases are caused by HPV-16 and approximately 3% each by HPV-18, HPV-33, and HPV-35 (70). Clinically, compared with cancers etiologically associated with tobacco and alcohol, HPV-AOC has a substantially better prognosis and superior response to treatment (13). Regarding differences at the molecular level, the carcinogenesis of HPV-AOC is driven mainly by HPV overexpressed proteins interacting with host tumor-suppressor genes. The HPV oncoprotein E6 inhibits the host tumor-suppressor gene p53, and the HPV oncoprotein E7 binds to the host tumor-suppressor gene pRb, promoting its degradation (13). Overexpression of p16^INK4a^, a tumor suppressor which is upregulated by high-level expression of E7, is critical for cell survival in all HPV-related tumors, whereas it is frequently inactivated in HPV-independent tumors. Therefore, p16^INK4a^ overexpression is a surrogate marker for transcriptionally active HPV infection. In addition to the presence of HPV oncoviral proteins, the most common genetic changes in HPV-AOC are in the phosphoinositide-3-kinase (PI3K) pathway, particularly involving activating mutations and amplifications of the PIK3CA oncogene (71). The host genes TP53 and cyclin-dependent kinase inhibitor 2A (CDKN2A)—most frequently affected in HPV-independent head and neck cancers, including oropharyngeal cancer—are largely unaffected in HPV-AOC (71).

The differences described between HPV-AOC and its counterpart associated with tobacco and alcohol have recently led to a new classification of p16^INK4a^-positive HPV-AOC for the eighth edition of the *American Joint Committee on Cancer Staging Manual* (14), a substantial revision of diagnostic approaches for oropharyngeal cancer, and the launch of several clinical trials of deintensified treatment. A recent large international study on 7,654 patients with oropharyngeal cancer showed that detection of p16^INK4a^ overexpression should be complemented with testing for HPV DNA or HPV mRNA to reduce the risk of misclassification of HPV-non-attributable tumors (11). Specifically, sometimes tumors can be p16^INK4a^ positive in the absence of HPV infection and behave clinically as p16^INK4a^/HPV-double-negative tumors in terms of poorer survival, particularly compared with p16^INK4a^/HPV-double-positive cases (11). Because oropharyngeal cancers are often detected at an advanced stage with low tumor size but high nodal dissemination (spread of cancer into lymph nodes), timely and accurate differentiation of HPV-AOC from HPV-non-attributable oropharyngeal cancers by HPV testing of tumor tissue using reliable HPV tests is of utmost importance (Table 1).

### Natural history of HPV-AOC.

**FIG 3:**
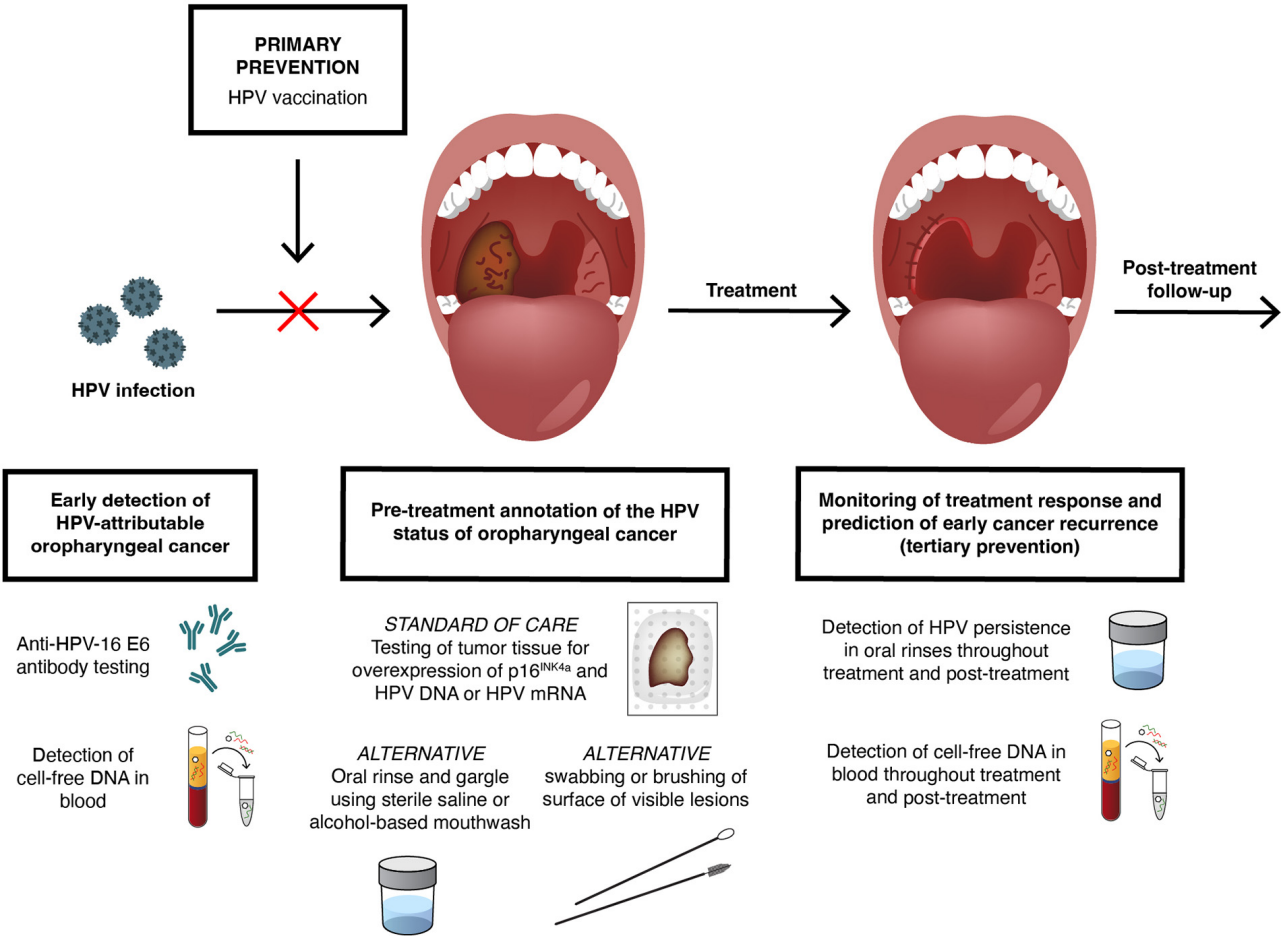
Primary, secondary, and tertiary prevention of HPV-attributable oropharyngeal cancer (HPV-AOC). Primary prevention of HPV-AOC by HPV vaccination might work for HPV-AOC, pending the results of a postapproval confirmatory study, which is still ongoing. No validated screening strategy for the secondary prevention of HPV-AOC aiming for early disease detection in the healthy population is available at present, but the following two approaches have been extensively explored in the last decade: (i) the detection of antibodies against HPV-16 E6 protein in blood and (ii) “liquid biopsies,” or the detection of cell-free DNA (including HPV DNA) in blood samples. Differentiation of HPV-AOC from HPV-non-attributable oropharyngeal cancers is currently a major clinical application of HPV testing in the field. Annotation of the HPV status of oropharyngeal cancer is best ascertained by testing tumor tissue (formalin-fixed, paraffin-embedded or fresh-frozen tissues) for overexpression of p16^INK4a^ using immunohistochemistry and the presence of HPV DNA or HPV mRNA by either *in situ* hybridization or PCR-based methods. Oral sample nontissue HPV-annotation alternatives, including (i) oral rinse and gargle using sterile saline or alcohol-based mouthwash and (ii) swabbing or brushing of surface of visible lesions, have been evaluated, and yet, the moderate sensitivity of these alternatives currently limits their use as diagnostics. In addition, nonoral alternatives, e.g., detection of cell-free DNA and antibodies against HPV-16 E6 protein in blood, have been also piloted for HPV-annotation purposes in selected clinical scenarios but with mixed and inconclusive results. The detection of HPV persistence in oral rinses and/or by detecting cell-free DNA in blood throughout treatment and posttreatment represent valid treatment response monitoring tools and promising predictive tools in the tertiary prevention of HPV-AOC (prevention of disease recurrences after definite therapy and/or prevention of the development of secondary HPV-AOC).

There are some similarities but also crucial differences in the natural history of HPV-AOC and HPV-related neoplasms in the anogenital context with important consequences for primary, secondary, and tertiary prevention approaches (Fig. 3). Similar to HPV-related anogenital disease, the first necessary step in the natural history of HPV-AOC is undoubtedly persistent HPV oral infection. In the general population, the prevalence of oral HPV infection with any HPV has been estimated to be around 5% and that for HPV-16 is around 1% (72). Regarding determinants of oral HPV infection, males have a higher prevalence than females, whereas the influence of age is not entirely clear (some studies show a bimodal distribution and others a slight increase of prevalence by age). Additionally, a history of smoking and sexual behavior has also been associated with oral HPV infection (73, 74). However, there remain knowledge gaps, and further research is needed to establish the incidence, clearance, and persistence rates of oral HPV infection, as well as its determinants, more precisely.

A small number of studies have explored the intraperson HPV genotype concordance (the same HPV genotype[s] present at different body sites) between anogenital and oral sites with conflicting results but generally showing low concordance. Also, intraperson hrHPV genotype concordance in males has been reported to be low, although it is higher in males who have sex with males than that in males who have sex with females (only) (75). Similar results have also been obtained in females, with hrHPV genotype concordance in anogenital and oral sites at around 5% only (76, 77). In practical terms, this information means that HPV status established in one body site is not a reliable indicator of the genotype(s) at other body sites. Site-specific HPV testing is therefore warranted.

The key knowledge gaps in the natural history of HPV-AOC that hinder the implementation of preventive measures similar to those used for decades for cervical cancer lie around the existence (or not) of HPV-related preneoplastic lesions that precede HPV-AOC and, if such lesions exist, whether they can be identified early enough to be treated in a safe, effective, and acceptable way to prevent the development of HPV-AOC, as in the case of cervical cancer (Fig. 3).

### Sampling oral cavity/oropharynx and testing for HPV.

Oral rinse and gargle using sterile saline or alcohol-based mouthwash are used most frequently for sampling the oral cavity or oropharynx for epidemiological, research, and clinical purposes (15, 78). The method used most frequently relies on 15- to 30-s rinsing followed by 15- to 30-s gargling (16, 79), although there is some heterogeneity in the literature. As is the case for first-void urine, evidence suggests that the use of preservative added immediately after sample capture can improve the stability of the oral sample for downstream molecular work (79). Oral samples should be processed promptly after the collection (especially in the absence of preservative) or frozen immediately at −20°C to minimize degradation (16). However, the impact of storage time (including at frozen temperatures) on sample quality/target deterioration remains unknown.

As an alternative to the rinse/gargle biospecimen, if oral lesions are visible, the mucosal surface has been sampled using a swab or brush. Saliva is not considered a robust specimen for HPV testing nor are specimens obtained by swabbing or brushing tonsils or the oropharynx without visible lesions (15, 78).

Relatively few studies have directly compared different sampling approaches in the oral cavity. One study compared oral gargles versus tonsil brushings in a sizeable age-stratified sample of oral/oropharyngeal cancer-free individuals and found that HPV detection was rare when using tonsil brushings from both children and adults (80). In contrast, HPV infection was detected more frequently in gargle specimens, particularly those of adults, but with poor agreement with the paired tonsillar sample (80).

In addition to obtaining a robust oral specimen, the choice of the appropriate test for detecting and genotyping HPV in oral samples is clearly important. Because HPV viral load in oral samples is significantly lower than that in genital sites, testing oral specimens requires the use of HPV tests with substantially higher analytical sensitivity (23). In the largest study to date to compare the performance of different HPV tests in oral specimens, 1,455 oral gargle samples from the HPV Infection in Men (HIM) study were tested in parallel using the Linear Array HPV genotyping test (Roche Molecular Systems, Alameda, CA) versus the SPF_10_ PCR-DEIA-LiPA_25_ system (DDL Diagnostic Laboratory, Rijswijk, Netherlands) with an overall HPV positivity of 1.9% versus 8.6%, respectively (81). The observed (significant) difference in HPV positivity is most likely a consequence of the substantially shorter region of HPV DNA targeted by SPF_10_ PCR-DEIA-LiPA_25_ (65 bp) than that of the Linear Array HPV genotyping test (approximately 450 bp). This difference in analytical performance becomes important in samples with very low viral load (such as gargle oral samples) or in samples with partially degraded DNA.

### Primary, secondary, and tertiary prevention of HPV-AOC.

Primary prevention by HPV vaccination might work equally well in the case of HPV-AOC as for anogenital HPV-related carcinomas but with a potential caveat. Namely, although the US FDA recently also conditionally approved an expanded indication of HPV vaccine for primary prevention of oropharyngeal and other head and neck cancers caused by targeted HPV genotypes, the final vaccine approval for this specific indication is dependent on the results of an ongoing postapproval confirmatory study (16).

As described above, no validated screening strategy for the secondary prevention of HPV-AOC to support early disease detection in the healthy population is routinely available at present (27). In contrast, promising results in the tertiary prevention of HPV-AOC were obtained recently (15). The tertiary prevention of HPV-AOC aims to prevent disease recurrence after definite therapy and/or to prevent the development of secondary HPV-AOC either by detecting cfDNA in blood (as presented above) or by detecting HPV in posttreatment oral rinses (as presented below).

### HPV testing of oral specimens in clinical practice.

Differentiation of HPV-AOC from HPV-non-attributable oropharyngeal cancers is currently a major clinical application of HPV testing in the field (27, 78). Annotation of the HPV status of oropharyngeal cancer is best ascertained by testing tumor tissue (fresh-frozen or formalin-fixed, paraffin-embedded tissue) for overexpression of p16^INK4a^ using immunohistochemistry and the presence of HPV DNA or HPV mRNA by either *in situ* hybridization or PCR-based methods (Fig. 3).

The use of nontissue alternatives for the annotation of the HPV status of oropharyngeal cancer has been studied intensively in the past decade. The accuracy of HPV testing in oral rinses or swabs for diagnosis of underlying HPV-AOC was assessed in a recent systematic review (82). Eight studies met the eligibility criteria, with five restricted to patients with oropharyngeal cancers. Five studies tested oral rinses, two studies used oral swabs, and in one study, all subjects had both an oral rinse and an oral swab. Seven studies used HPV DNA testing, and in one study, patients were tested for HPV-16 E6/E7 mRNA. In the five studies comprising oropharyngeal cancer patients, the sensitivity of oral HPV testing for detecting underlying HPV-AOC was 55% (95% CI, 25 to 82) with a specificity of 94% (95% CI, 85 to 98). Overall sensitivity across all eight eligible studies was 72% (95% CI, 82 to 97) but with considerable variability in the sensitivity estimates ranging from 93% (95% CI, 81 to 99) to only 12% (95% CI, 7 to 19) (82).

The moderate sensitivity of HPV testing in oral rinses at HPV-AOC diagnosis limits its use as a screening test for HPV-AOC. However, akin to liquid biopsy, HPV testing in oral rinses could be used as an adjunct prognostic marker in the tertiary prevention of HPV-AOC, where the persistence of the HPV in oral rinses throughout treatment may predict HPV-AOC recurrences and/or the development of secondary HPV-AOC (Fig. 3). Rettig et al. (83) detected baseline oral HPV-16 DNA infection in 67/124 (54%) of patients diagnosed with HPV-AOC with only five patients having persistent HPV-16 infection in oral rinses at 24 months after treatment. All five patients (100%) with persistent HPV-16 infection (HPV-16 present at baseline and after therapy) developed recurrent disease, with three with local disease involvement. In contrast, 9 out of 119 (8%) patients without persistent HPV-16 infection developed recurrent disease (83). Two similar but smaller studies using oral rinses showed comparable results (84, 85), with a baseline cancer detection rate of HPV-AOC at around 50% and significantly higher recurrence rates in patients with persistent oral HPV-16 infection versus those without, namely, 100% versus 11% (84) and 75% versus 6% (85), respectively. These data suggest that, although infrequent, persistent HPV-16 infection detected in posttreatment oral rinses is associated with a poor prognosis of HPV-AOC and represents a potential marker for long-term tumor surveillance, mainly for the early detection of recurrent HPV-AOC with local disease involvement.

## SUMMARY

Testing for hrHPV using clinically validated molecular HPV assays has dramatically changed our ability to screen for cervical cancer, gradually replacing traditional morphology-based screening using the Pap test. In addition to clinician-collected cervical samples and self-collected cervicovaginal samples, first-void urine is emerging as a credible specimen for HPV-based cervical cancer screening, triage of HPV screen-positive women, monitoring of HPV vaccine impact, and HPV testing in groups where a less invasive sample is preferable (including transgender individuals). Detection of cell-free DNA (including HPV DNA) in blood samples is slowly transitioning into routine practice, holding great promise for various applications, including the early detection of HPV-attributable oropharyngeal cancer (HPV-AOC) and potentially other anogenital cancers, as well as an adjunct prognostic marker in long-term tumor surveillance. Anti-HPV-16 E6 antibody presence in blood may serve as a potential screening, diagnostic, and prognostic marker of HPV-AOC, but greater research in this area is warranted. The moderate sensitivity of HPV testing in oral rinses or swabs at HPV-AOC diagnosis prevents its use as a screening or diagnostic tool, but HPV testing in oral rinses represents a promising prognostic tool in the tertiary prevention of HPV-AOC, where persistence in oral rinses through and after treatment may predict early HPV-AOC recurrences and/or the development of secondary HPV-AOC. In addition, HPV testing of oral rinses will continue to be important for natural history and epidemiological studies.

In conclusion, three specimen types traditionally considered “alternative” for HPV testing (urine, blood, and oral specimens) are shown to have an increasing clinical, epidemiological, and scientific value (Table 1). The HPV community should work together to identify, report, and optimize all key technical steps (from sample collection to final result) and to make recommendations for best laboratory and clinical practice. Thus, we can gain a more accurate insight into the true benefits of application when the whole testing workflow is optimized and validated.
